# Disparities in time-to-treatment initiation and therapeutic modalities for malignant melanoma of the skin: a cross-sectional study, Brazil, 2013-2023

**DOI:** 10.1590/S2237-96222026v35e20250025.en

**Published:** 2026-02-06

**Authors:** Helena Rubini Nogueira, Enzo Lofredo Amorim, Geovanna Dhi Genaro, Múcio Cevulla da Silva Lourenço, Victor Hugo Silva Dantas, Luiz Vinicius de Alcantara Sousa

**Affiliations:** 1 Universidade Municipal de São Caetano do Sul, São Caetano do Sul, SP, Brazil

**Keywords:** Brazil, Epidemiology, Melanoma, Cutaneous Malignant, Public Health, Time-to-Treatment, Brasil, Epidemiología, Melanoma Cutáneo Maligno, Salud Pública, Tiempo de Tratamiento

## Abstract

**Objective:**

To analyze regional disparities in time-to-treatment initiation and initial therapeutic modalities adopted for malignant melanoma of the skin in Brazil between 2013 and 2023.

**Method:**

This is a cross-sectional study based on secondary data extracted from the Oncology Panel. Prevalence ratios and 95% confidence intervals for time-to-treatment initiation were estimated by treatment region and initial therapeutic modality using Poisson regression with robust variance.

**Results:**

A total of 23,145 records were analyzed. Surgery was the main initial therapeutic modality in all regions, with 89.0% of procedures performed within 30 days of diagnosis, especially in the Southern region (88.6%). In contrast, chemotherapy and radiotherapy were predominantly initiated after 60 days, with the highest percentages in the Northern region (56.1% and 77.4%).

**Conclusion:**

Significant disparities in time-to-treatment initiation were observed between the Brazilian regions, regardless of therapeutic modality, except in the Midwest region regarding radiotherapy. The findings highlighted the need for actions to promote greater equity in access to cancer treatment, in accordance with Law No. 12732/2012, aiming to improve clinical outcomes for users.

## Resumen

**Objetivo:**

Analizar las disparidades regionales en el tiempo de inicio del tratamiento y las modalidades terapéuticas iniciales adoptadas para el melanoma maligno de piel en Brasil entre 2013 y 2023.

**Método:**

Estudio transversal basado en datos secundarios extraídos del Panel de Oncología. Se estimaron las razones de prevalencia y los intervalos de confianza del 95% para el tiempo de inicio del tratamiento por región de tratamiento y modalidad terapéutica inicial mediante regresión de Poisson con varianza robusta.

**Resultados:**

Se analizaron 23.145 registros. La cirugía fue la principal modalidad terapéutica inicial en todas las regiones, con el 89,0% de los procedimientos realizados dentro de los 30 días posteriores al diagnóstico, especialmente en la región Sur (88,6%). Por el contrario, la quimioterapia y la radioterapia se iniciaron predominantemente después de los 60 días, con los porcentajes más altos en la región Norte (56,1% y 77,4%).

**Conclusión:**

Se observaron disparidades significativas en el tiempo de inicio del tratamiento en las distintas regiones brasileñas, independientemente de la modalidad terapéutica, excepto en la región Centro-Oeste en lo que respecta a la radioterapia. Los hallazgos destacaron la necesidad de acciones para promover una mayor equidad en el acceso al tratamiento del cáncer, de conformidad con la Ley n.º 12.732/2012, con el objetivo de mejorar los resultados clínicos de los usuarios.

**Ethical aspects**

This research used public domain anonymized databases.:

## Introduction

Malignant melanoma of the skin is a type of cancer that has been showing high incidence and mortality rates since the 1990s. Compared to 2012, 2020 saw a 41% increase in the number of cases worldwide, rising from 230,000 to 325,000 cases (1). Carcinogenesis occurs through mutations in melanocytic cells, the main function of which is to produce melanin. This process is due to genetic, hereditary and environmental factors, with particular emphasis on exposure to ultraviolet rays (2).

Diagnosis of cutaneous melanoma initially involves an excisional biopsy followed by anatomopathological analysis to determine tumor staging. This analysis considers criteria such as: Breslow thickness; presence of ulceration; dermal mitotic rate; margin status; presence of microsatellites; desmoplasia; and lymphovascular invasion (3,4,5).

Treatment options include surgery to remove the tumor with wide margins, sentinel lymph node investigation, and adjuvant therapies such as radiotherapy, immunotherapy and chemotherapy (6,7). Other factors may compromise treatment effectiveness and equity, such as unavailability of immunotherapies, limited access to molecular testing, regional limitations and social factors that compromise treatment effectiveness and equity (8).

Surgical treatment is the first option, as most people with this neoplasm present it in early stages IA (T1a, N0, M0) and IIA (T2a or T3a, N0, M0), both in Brazil and in developed countries, providing a cure rate of 70% to 90% of cases. This option is not indicated *a priori* in the context of disseminated stage IV (unresectable) or for those receiving palliative care. After excisional biopsy, wide excision of the residual scar is performed, with widening of the margins according to clinical staging, due to the frequency of satellite lesions that can result in recurrence (9).

Use of radiotherapy as a treatment method in this scenario involves emission of ionizing radiation to prevent the development of carcinoma. This method is prioritized when surgery is impossible or in cases of high risk of recurrence. The choice of radiotherapy depends on analysis of the following factors: lesion size, location and depth of infiltration, team experience and institutional resources (10).

Immunotherapy involves stimulating a person’s own immune system to recognize and fight cancer; this technique has revolutionized the treatment of cutaneous melanoma. Advances in understanding tumor evasion mechanisms have led to the development of immune checkpoint inhibitors, intravenously administered monoclonal antibodies that have become essential for both adjuvant treatment (for people with positive lymph nodes) and metastatic treatment (11).

Use of chemotherapy to treat melanoma is reserved for metastatic cases, usually after failure of treatments such as immunotherapy or targeted therapy. Chemotherapy protocols may include a single drug or a combination of several, administered intravenously or orally through tablets and capsules (11). Although this modality has not been shown to significantly increase overall survival in people with melanoma, it can offer symptom relief, therapeutic responses and prolong the time to recurrence in some cases (12).

The importance of regular monitoring to detect recurrences and adjust treatment according to stage and initial response is emphasized (1,2). However, waiting time to treatment initiation is also decisive for the course of disease development and intervention strategies. As established by Law No. 12732/2012 (13), in Brazil there is a 60-day deadline for cancer treatment initiation, with effect from the diagnosis based on an anatomopathological report.

This text aimed to analyze the disparities between time-to-treatment initiation and the therapeutic modalities chosen for health service users with malignant melanoma of the skin in the different Brazilian regions from 2013 to 2023.

## Methods

### Design

This is an observational cross-sectional study of cases of malignant melanoma of the skin reported in the Brazilian regions from 2013 to 2023.

### Setting

Law No. 12732/2012 established a 60-day deadline for starting oncological treatment based on a confirmatory anatomopathological report, in order to guarantee better survival rates and increase the chances of positive prognosis (13).

### Participants

All records of people diagnosed with malignant melanoma of the skin (code C43 of the 10^th^ revision of the International Classification of Diseases – ICD-10) held on the Oncology Panel between 2013 and 2023 were included.

### Variables

The data were stratified according to the following variables.

- 070 Malignant neoplasm of the skin: the term C43 was chosen, which corresponds specifically to malignant melanoma of the skin, excluding other neoplasms that may affect the integumentary system.
- Time-to-treatment: interval between the date of the diagnostic examination and the date of the first treatment calculated in days and divided into 0-30 days, 31-60 days, more than 60 days and no treatment information.
- Therapeutic modality: procedure used for first treatment, which may be surgery, chemotherapy, radiotherapy or both (chemotherapy and radiotherapy with the same treatment date).
- Treatment regions: region in which the health service user was treated, classified as North, Northeast, Midwest, Southeast and South.
- Period: from 2013 to 2023.

We chose to present the information in the Results section cross-sectionally by geographic region and therapeutic modality, thus enabling distribution of cases by treatment type in each region of Brazil. The number of cases treated with surgery, chemotherapy, radiotherapy and a combination of the latter two methods was specified for each region (North, Northeast, Midwest, Southeast and South), thus facilitating understanding of regional disparities in access to treatment.

### Data sources 

The data were obtained through the Oncology Panel, a Ministry of Health tool processed by the Brazilian Unified Health System’s Information Technology Department. In this panel, data integration occurred by matching National Health Card data with the ICD-10 code, taken from the Outpatient Information System, the Hospital Information System and the Cancer Information System. 

The data used were accessed between January and February 2024, covering the period 2013-2023. The database was deposited in a public repository with open access: https://osf.io/np6b8/ (14).

### Bias 

The main risks of bias relate to possible underreporting or inconsistencies in data matching between the information systems that provide the data extracted from the Oncology Panel. To mitigate this bias, all available data were used, ensuring comprehensiveness and representativeness. However, limitations such as absence of clinical variables, such as tumor staging and progression, may compromise complete control of confounding factors.

### Statistical methods 

The data were analyzed using absolute and relative values ​​to describe the distribution of time to cancer treatment initiation according to therapeutic modality, from 2013 to 2023. To estimate association between Brazilian regions and the time elapsed to treatment initiation, we used a Poisson regression model with robust variance, stratified by the first therapeutic modality administered. This approach was chosen because it is more appropriate for cross-sectional studies with binary outcomes and high prevalence, allowing for direct calculation of the prevalence ratio, a more interpretable and accurate measure than the odds ratio, which tends to overestimate association in scenarios with high frequency of the outcome.

The models were adjusted for potential confounding factors, taking a 95% confidence level. Statistical analyses were conducted using Stata (Data Analysis and Statistical Software for Professionals), version 16.0.

## Results

We quantified the different therapeutic modalities performed based on the selected sample of 23,145 records, encompassing all types, and times-to-treatment initiation (Table 1). 12,070 people underwent surgery; 1,076 (9.0%) did not undergo surgery within 60 days.

**Table 1:** Frequency and percentage of time-to-treatment initiation, by therapeutic modality. Brazil, 2013-2023 (n=23,145)

Variable	n (%)
**Surgery** (days)	12,070 (100.0)
≤30	10,716 (89.0)
31-60	278 (2.0)
≥60	1,076 (9.0)
**Chemotherapy** (days)	8,538 (100.0)
≤30	1,618 (19.0)
31-60	1,383 (16.0)
≥60	5,537 (65.0)
**Radiotherapy** (days)	2,523 (100.0)
≤30	287 (11.0)
31-60	450 (18.0)
≥60	1,786 (71.0)
**Chemotherapy and radiotherapy** (days)	14 (100.0)
≤30	4 (29.0)
31-60	2 (14.0)
≥60	8 (57.0)

A total of 8,538 service users underwent chemotherapy, of whom 5,537 (65.0%) had access to this therapeutic modality later than the 60-day limit. In the case of the 2,523 people who underwent radiotherapy, the records showed that 1,786 (71.0%) also had their sessions after the 60-day limit (Table 1).

Of the total of 14 people who were referred for both treatments, i.e. concomitant chemotherapy and radiotherapy, eight (57.0%) underwent them after the 60-day limit. Finally, there was no information on treatment and the time it took to start it after diagnosis for 13,403 service users (Table 1).

We sought to gain understanding of the time-to-treatment initiation prevalence ratio, according to the treatment region and the first therapeutic modality performed, from 2013 to 2023 for the same sample (Table 2). There was a significant difference regarding treatment initiation in the regions studied (p-value<0.050), regardless of the therapeutic modality performed, with the exception of the Midwest region with regard to radiotherapy (p-value 0.060).

**Table 2:** Analysis of the prevalence ratio (PR) of time-to-treatment initiation, by treatment region and first therapeutic modality performed. Brazil, 2013-2023 (n=23,145)

Region	North			Northeast			Southeast			South			Midwest		
	%	PR	p-value	%	PR	p-value	%	PR	p-value	%	PR	p-value	%	PR	p-value
**Surgery** (days)			<0.001			<0.001			<0.001			<0.001			0.007
≤30	78.2	Ref.^a^		88.6	Ref.		82.5	Ref.		88.6	Ref.		85.9	Ref.	
31-60	3.3	23.42		2.8	31.38		3.5	23.03		2.7	31.78		3.0	28.73	
≥60	18.4	4.25		8.5	10.39		13.8	5.96		8.5	10.37		11.0	7.77	
**Chemotherapy** (days)			<0.001			0.027			0.033			<0.001			0.001
≤30	23.1	Ref.		26.3	Ref.		23.7	Ref.		33.5	Ref.		27.1	Ref.	
31-60	20.7	1.11		23.4	1.12		22.8	1.04		24.4	1.37		24.3	1.11	
≥60	56.1	0.41		50.2	0.52		53.4	0.44		42.0	0.80		48.6	0.56	
**Radiotherapy** (days)			0.001			<0.001			0.023			0.003			0.060
≤30	9.5	Ref.		11.6	Ref.		11.4	Ref.		15.1	Ref.		10.1	Ref.	
31-60	13.0	0.73		18.3	0.63		15.7	0.73		20.2	0.75		15.0	0.67	
≥60	77.4	0.12		70.0	0.17		72.8	0.16		64.6	0.23		74.9	0.13	

^a^Ref.=reference.

The five regions followed the same patterns when the first treatment was surgery, with the highest percentage occurring within 30 days of diagnosis (89.0%), whereby the prevalence ratio of intervention within 30 days of diagnosis was higher than the other time intervals. The Southern region stood out the most, with 88.6% of surgeries occurring within 30 days of diagnosis. The Northern region had the lowest percentage (78.2%) of surgeries performed before 30 days (Table 2).

When comparing treatment region and choice of chemotherapy, contrary to what was observed with the “surgery” variable, the highest percentage of these interventions occurred after 60 days (65.0%), whereby the prevalence ratio of this interval was higher than the others. The Northern region had the highest percentage of chemotherapy treatment (56.1%) performed after 60 days of diagnosis, and the lowest percentage (23.1%) performed before 30 days (Table 2).

Seventy-one percent of radiotherapies were performed more than 60 days after diagnosis, so that the prevalence ratio for this interval was higher than the others. The Northern region had the highest percentage performed after 60 days (77.4%) and the lowest number of sessions performed up to 30 days after diagnosis (9.5%) (Table 2).

Although some delays in treatment initiation are evident, it is not possible to cross-reference the data to relate them to other variables to determine whether the late start of treatments was due to presence of metastasis, availability of treatment, or other clinical and social factors.

## Discussion

The majority of the Brazilian population, as required by law, had access to treatment for malignant melanoma of the skin within less than 30 days of confirmed diagnosis. However, significant regional inequalities in the time-to-treatment initiation were observed, mostly regardless of the initial therapeutic modality.

In this context, surgery was the primary treatment option indicated in all regions and presented the shortest intervals between diagnosis and treatment. The Northern region stood out due to the predominance of late treatment initiation, especially when the initial indication was chemotherapy and radiotherapy.

Among the limitations of this research, we highlight the use of anonymized secondary data derived from the matching of information systems data consolidated in the Oncology Panel records. This prevented access to detailed clinical variables, such as tumor staging, comorbidities, histological type and clinical outcomes.

There is a possibility of underreporting and inconsistencies in records, in addition to the exclusive study of cases from the Brazilian Unified Health System (*Sistema* Único *de Saúde*, SUS), fostering greater selection bias. Despite this, the nationwide coverage, periodic updates and robust time series strengthen the analysis in support of addressing health inequalities.

Delays in initiating cancer treatment are associated with poorer prognosis, more complications and shorter survival, especially in the case of neoplasms with metastatic potential such as melanoma (15,16,17). Therefore, it is important to question the reasons that lead to regional differences in health care initiation, as they may reflect inequalities in access to specialized services, oncological infrastructure and the effectiveness of cancer treatment regulation across the country (18).

A crucial aspect in this context is coordination between different specialists in order to avoid delays in melanoma treatment. An example of this is people who undergo biopsy and excision procedures with the same dermatologist and, consequently, experience fewer delays in their therapeutic care (19). 

In the United States, for example, there is no formal recommendation specifying a timeline from biopsy to definitive treatment (20,21). In Europe, however, there is consensus to initiate surgical treatment within four to six weeks simultaneously with sentinel lymph node biopsy (22), which corroborates the findings of higher prevalence of surgery within 30 days in Brazil.

Depending on the stage of the disease, adjuvant treatments, such as immunotherapy, are chosen (23,24,25). Since 2010, new systemic treatments have been approved in the context of cutaneous malignant melanoma: systemic immunotherapy, single-agent B-Raf (BRAF) proto-oncogene inhibitors, BRAF-MEK inhibitor combination regimens, and intralesional immunotherapy involving a modified oncolytic herpes virus (26).

These treatments were initially indicated for advanced and unresectable melanoma (26). However, it was found that these new strategies offer better efficacy when compared to chemotherapy. Despite this information, chemotherapy and immunotherapeutics of the anti-PD-1 class (nivolumab and pembrolizumab) remain the only systemic therapies offered by the Brazilian Unified Health System in the treatment of this type of cancer, with dacarbazine being the main chemotherapeutic agent used (27).

In Canada (28), 302 individuals diagnosed with stage IV cutaneous melanoma with metastasis present at diagnosis were analyzed. In that population, the median time to initiation of immunotherapy as first-line treatment was 52 days (interquartile range 30.2–99.0 days). As of 2025, no other studies reporting the use of alternative systemic therapies as the initial choice in this clinical setting were found.

There is also the possibility of combining chemotherapy with radiotherapy. This can occur sequentially or concomitantly, based on the assumption that administration of concomitant chemotherapy ensures better local control of melanoma, thus optimizing the results of radiotherapy (20).

It can be inferred that, once other systemic therapies are included in the repertoire of treatments available through the Brazilian Unified Health System, the survival results for stage IV melanoma will be more promising than those with the exclusive use of chemotherapy (20).

Regarding targeted therapy, melanoma is known to be a relatively radioresistant tumor, as it has the ability to effectively repair DNA damage caused by radiation (29). Therefore, radiotherapy is chosen as a first-line treatment in exceptional cases—for example, when surgery is impossible or as a complement in certain situations where there is a high risk of recurrence. On the other hand, this therapeutic modality is widely used as palliative treatment for metastatic melanoma (stage IV).

The high prevalence of individuals who underwent radiotherapy later than 60 days after diagnosis can be justified by the assumption that they were identified in an advanced stage of the disease, in which treatment has a palliative purpose, seeking to alleviate symptoms and prolong local control of the tumor (29).

The findings of this study indicated significant disparities in the initiation of cancer treatment for malignant melanoma of the skin in Brazil, with surgery being the most timely and consistent procedure across regions. Chemotherapy and radiotherapy presented later initiation and greater regional variation, suggesting barriers to access and possible delays in care. The findings also highlight the need for further studies on investment in immunotherapy in Public Health. This evidence reinforces the need for public policies that ensure compliance with current legislation and expanded access to more modern and effective therapies within the Brazilian Unified Health System.

## Data Availability

The database used in this research is available at: https://osf.io/np6b8/.
