# Supplementary material for: Disparities in time-to-treatment initiation and therapeutic modalities for malignant melanoma of the skin: a cross-sectional study, Brazil, 2013-2023
Source: Epidemiol Serv Saude. 2026 Feb 6;35:e20250025. doi: 10.1590/S2237-96222026v35e20250025.en (PMC12928547; doi:10.1590/S2237-96222026v35e20250025.en)
Supplement: Translation

# Translation: Disparidades no tempo para início do tratamento e nas modalidades terapêuticas para melanoma maligno de pele: estudo transversal, Brasil, 2013-2023

**DOI:** 10.1590/S2237-96222026v35e20250025.pt

**Authors:** Helena Rubini Nogueira, Enzo Lofredo Amorim, Geovanna Dhi Genaro, Múcio Cevulla da Silva Lourenço, Victor Hugo Silva Dantas, Luiz Vinicius de Alcantara Sousa

**Affiliations:** 1 Universidade Municipal de São Caetano do Sul, São Caetano do Sul, SP, Brasil

**Keywords:** Brasil, Epidemiologia, Melanoma Maligno Cutâneo, Saúde Coletiva, Tempo para o Tratamento

## Abstract

**Objetivo:**

Analisar as disparidades regionais no tempo para início do tratamento e nas modalidades terapêuticas iniciais adotadas para o melanoma maligno de pele no Brasil entre 2013 e 2023.

**Método:**

Trata-se de estudo transversal baseado em dados secundários extraídos do Painel-Oncologia. Estimaram-se razões de prevalência e intervalos de confiança de 95% para o tempo até o início do tratamento, segundo a região de tratamento e a modalidade terapêutica inicial, utilizando-se regressão de Poisson com variância robusta.

**Resultados:**

Foram analisados 23.145 registros. A cirurgia foi a principal modalidade terapêutica inicial em todas as regiões, com 89,0% dos procedimentos realizados em até 30 dias após o diagnóstico, especialmente na região Sul (88,6%). Em contraste, a quimioterapia e a radioterapia foram predominantemente iniciadas após 60 dias, com os maiores percentuais na região Norte (56,1% e 77,4%).

**Conclusão:**

Evidenciaram-se disparidades significativas no tempo para início do tratamento entre as regiões brasileiras, independentemente da modalidade terapêutica, exceto no Centro-Oeste em relação à radioterapia. Os achados ressaltaram a necessidade de ações que promovam maior equidade no acesso ao tratamento oncológico, em conformidade com a Lei nº 12.732/2012, visando à melhoria dos desfechos clínicos dos usuários.

**Aspectos éticos**

Esta pesquisa utilizou bancos de dados de domínio público e anonimizados.:

## Introdução

O melanoma maligno de pele é um tipo de câncer que vem apresentando elevadas taxas de incidência e mortalidade desde a década de 1990. Em comparação com 2012, o ano de 2020 apresentou aumento mundial do número de casos de 41%, migrando de 230 mil para 325 mil casos (1). No que diz respeito à sua carcinogênese, esta ocorre por mutações das células melanocíticas, cuja principal função é produzir a melanina, sendo esse advento decorrente de fatores genéticos, hereditários e ambientais, ressaltando-se aqui a exposição aos raios ultravioleta (2).

O diagnóstico de melanoma cutâneo envolve inicialmente a realização de biópsia excisional seguida de análise anatomopatológica para determinar o estadiamento tumoral. Nessa análise, são considerados critérios como: espessura de Breslow; presença de ulceração; taxa mitótica dermal; status das margens; presença de microssatélites; desmoplasia; e invasão linfovascular (3,4,5). 

As opções de tratamento incluem cirurgia para remoção do tumor com margens ampliadas, investigação de linfonodo sentinela e terapias adjuvantes como radioterapia, imunoterapia e quimioterapia (6,7). Outros fatores podem comprometer a efetividade do tratamento e sua equidade, como a indisponibilidade de imunoterapias, o acesso restrito a testes moleculares, as limitações regionais e os fatores sociais que comprometem a efetividade do tratamento e sua equidade (8).

O tratamento cirúrgico é a primeira opção, visto que a maioria das pessoas com a neoplasia a apresenta em estádios iniciais IA (T1a, N0, M0) e IIA (T2a ou T3a, N0, M0), tanto no Brasil quanto em países desenvolvidos, o que representa a taxa de cura de 70% a 90% dos casos. Essa opção apenas não é indicada a priori no contexto de estádio IV disseminado (irressecável) e para aqueles em cuidados paliativos. Posteriormente à biópsia excisional, procede-se à excisão ampla da cicatriz residual, com ampliação das margens conforme o estadiamento clínico, devido à frequência de lesões satélites que podem resultar em recidiva (9).

A utilização da radioterapia como método de tratamento nesse cenário consiste na emissão de radiações ionizantes, a fim de evitar o desenvolvimento do carcinoma, sendo o método priorizado na impossibilidade cirúrgica ou em casos de grande risco de recidiva. Sua escolha depende da análise dos seguintes fatores: tamanho da lesão, localização e profundidade da infiltração, experiência da equipe e recursos institucionais (10).

A imunoterapia ocorre a partir da estimulação do próprio sistema imunológico da pessoa para reconhecer e combater o câncer; essa técnica revolucionou o tratamento do melanoma cutâneo. O avanço no entendimento dos mecanismos de evasão tumoral permitiu o desenvolvimento dos inibidores de pontos de verificação imunológicos, anticorpos monoclonais administrados por via intravenosa que se tornaram fundamentais no tratamento tanto adjuvante (em pessoas com linfonodos positivos) quanto metastático (11).

O uso da quimioterapia no melanoma é reservado para casos metastáticos, geralmente após falha de tratamentos como a imunoterapia ou a terapia-alvo. Os protocolos quimioterápicos podem incluir um único medicamento ou uma combinação de vários, administrados via intravenosa ou oral por meio de comprimidos e cápsulas (11). Embora essa modalidade não tenha demonstrado aumento significativo na sobrevida global de pessoas com melanoma, ela pode oferecer alívio dos sintomas, respostas terapêuticas e prolongamento do tempo até a recorrência em alguns dos casos (12).

Ressalta-se a importância do acompanhamento regular para detecção de recidivas e ajuste do tratamento conforme estágio e resposta inicial (1,2). Porém, também determinante para o curso do desenvolvimento da doença e das estratégias de intervenção, está o tempo de espera para o início do tratamento. Como destacado pela Lei nº 12.732/2012 (13), no Brasil existe o prazo de 60 dias para início do tratamento oncológico, a partir do diagnóstico pelo laudo anatomopatológico. 

Este texto teve como objetivo analisar as disparidades entre o tempo para início do tratamento e as modalidades terapêuticas escolhidas para o usuário com melanoma maligno de pele nas diferentes regiões brasileiras de 2013 a 2023. 

## Métodos

### Delineamento do estudo

Trata-se de estudo observacional, de caráter transversal a respeito dos casos de melanoma maligno de pele notificados nas regiões brasileiras de 2013 a 2023.

### Contexto

De acordo com a Lei nº 12.732/2012, definiu-se o prazo de 60 dias para o início de tratamento oncológico pelo laudo anatomopatológico confirmatório, a fim de garantir melhor sobrevida às pessoas e aumentar as chances de um prognóstico positivo (13).

### Participantes

Foram incluídos todos os registros de pessoas com diagnóstico de melanoma maligno de pele (C43, com base na 10ª revisão da Classificação Internacional de Doenças – CID-10) disponíveis no Painel-Oncologia entre 2013 e 2023.

### Variáveis

Os dados foram estratificados de acordo com as variáveis a seguir.

- 070 Neoplasia maligna da pele: optou-se pelo uso da denominação C43, que corresponde especificamente ao melanoma maligno da pele, excluindo outras neoplasias que possam afetar o sistema tegumentar.
- Tempo de tratamento: intervalo entre a data do exame diagnóstico e a data do primeiro tratamento calculado em dias e dividido em 0-30 dias, 31-60 dias, mais de 60 dias e sem informação de tratamento.
- Modalidade terapêutica: procedimento condizente com o primeiro tratamento, podendo ser cirurgia, quimioterapia, radioterapia e ambos (quimioterapia e radioterapia com a mesma data de tratamento).
- Regiões de tratamento: região em que usuário foi tratado, classificadas em Norte, Nordeste, Centro-Oeste, Sudeste e Sul.
- Período: de 2013 a 2023.

Optou-se pela exposição das informações nos Resultados, de forma cruzada, por região geográfica e modalidade terapêutica, o que permitiu observar a distribuição de casos por tipo de tratamento em cada região do Brasil. O número de casos tratados com cirurgia, quimioterapia, radioterapia e combinação dos dois métodos foi especificado para cada região (Norte, Nordeste, Centro-Oeste, Sudeste e Sul), o que facilitou a compreensão das disparidades regionais no acesso ao tratamento.

### Fontes de dados

Os dados foram obtidos por meio do Painel‑Oncologia, ferramenta do Ministério da Saúde processada pelo Departamento de Informática do Sistema Único de Saúde. Nesse painel, a integração dos dados ocorreu via pareamento dos dados pelo Cartão Nacional de Saúde com o CID‑10, provenientes do Sistema de Informações Ambulatoriais, do Sistema de Informações Hospitalares e do Sistema de Informações de Câncer. 

O acesso aos dados utilizados ocorreu entre janeiro e fevereiro de 2024, considerando o período 2013-2023. O banco de dados construído foi depositado em repositório público com acesso aberto: https://osf.io/np6b8/ (14).

### Viés

Os principais riscos de viés referem-se à possível subnotificação ou inconsistências de pareamento dos dados entre os sistemas de informação que fornecem os dados retirados do Painel-Oncologia. Para mitigar esse viés, foram utilizados todos os dados disponíveis, garantindo abrangência e representatividade. Ainda assim, limitações como ausência de variáveis clínicas, como o estadiamento tumoral e a evolução, podem comprometer o controle completo de fatores de confusão.

### Métodos estatísticos

As informações foram analisadas por meio de valores absolutos e relativos, com o objetivo de descrever a distribuição do tempo para início do tratamento oncológico segundo a modalidade terapêutica, no período 2013-2023. Para estimar a associação entre as regiões brasileiras e o tempo decorrido até o início do tratamento, estratificado pela primeira modalidade terapêutica realizada, utilizou-se o modelo de regressão de Poisson com variância robusta. Essa abordagem foi escolhida por ser mais apropriada em estudos transversais com desfechos binários e elevada prevalência, o que permitiu a obtenção direta da razão de prevalência, medida mais interpretável e acurada do que a razão de chances, que tende a superestimar a associação em cenários de alta frequência do desfecho. 

Os modelos foram ajustados para potenciais fatores de confusão, considerando o nível de confiança de 95%. As análises estatísticas foram conduzidas utilizando o software Stata (Data Analysis and Statistical Software for Professionals), versão 16.0.

## Resultados

Observou-se a quantificação entre as modalidades terapêuticas realizadas pela amostra selecionada de 23.145 registros, englobando todos os gêneros, e os tempos para início do tratamento (Tabela 1). Foram submetidas 12.070 pessoas à cirurgia; 1.076 (9,0%) das pessoas não operaram no período de 60 dias. 

**Tabela 1:** Frequência e percentual do tempo para início do tratamento, segundo a modalidade terapêutica. Brasil, 2013-2023 (n=23.145)

Variável	n (%)
**Cirurgia** (dias)	12.070 (100,0)
≤30	10.716 (89,0)
31-60	278 (2,0)
≥60	1.076 (9,0)
**Quimioterapia** (dias)	8.538 (100,0)
≤30	1.618 (19,0)
31-60	1.383 (16,0)
≥60	5.537 (65,0)
**Radioterapia** (dias)	2.523 (100,0)
≤30	287 (11,0)
31-60	450 (18,0)
≥60	1.786 (71,0)
**Quimioterapia e radioterapia** (dias)	14 (100,0)
≤30	4 (29,0)
31-60	2 (14,0)
≥60	8 (57,0)

Passaram por quimioterapia 8.538 usuários, dos quais 5.537 (65,0%) tiveram acesso a essa modalidade terapêutica apenas após os 60 dias. No caso das 2.523 pessoas que se submeteram à radioterapia, contabilizou-se que 1.786 (71,0%) passaram pelas sessões, também após o limite dos 60 dias (Tabela 1). 

Quanto a ambos os tratamentos, quimioterapia e radioterapia concomitantes, do total de 14 pessoas que foram direcionadas, oito (57,0%) as realizaram após os 60 dias. Por fim, 13.403 não possuíam informações de tratamento e tempo que levaram para iniciá-lo após o diagnóstico (Tabela 1). 

Buscou-se o entendimento acerca da razão de prevalência do tempo para início do tratamento, segundo a região de tratamento e a primeira modalidade terapêutica realizada, de 2013 a 2023 da mesma amostra (Tabela 2). Houve diferença significativa quanto ao início de tratamento nas regiões estudadas (p-valor<0,050), independentemente da modalidade terapêutica realizada, com exceção do Centro-Oeste no que concerne à radioterapia (p-valor 0,060).

**Tabela 2:** Análise da razão de prevalência (RP) do tempo para início do tratamento, segundo a região de tratamento e a primeira modalidade terapêutica realizada. Brasil, 2013-2023 (n=23.145)

Região	Norte			Nordeste			Sudeste			Sul			Centro-Oeste		
	%	RP	p-valor	%	RP	p-valor	%	RP	p-valor	%	RP	p-valor	%	RP	p-valor
**Cirurgia** (dias)			<0,001			<0,001			<0,001			<0,001			0,007
≤30	78,2	Ref.^a^		88,6	Ref.		82,5	Ref.		88,6	Ref.		85,9	Ref.	
31-60	3,3	23,42		2,8	31,38		3,5	23,03		2,7	31,78		3,0	28,73	
≥60	18,4	4,25		8,5	10,39		13,8	5,96		8,5	10,37		11,0	7,77	
**Quimioterapia** (dias)			<0,001			0,027			0,033			<0,001			0,001
≤30	23,1	Ref.		26,3	Ref.		23,7	Ref.		33,5	Ref.		27,1	Ref.	
31-60	20,7	1,11		23,4	1,12		22,8	1,04		24,4	1,37		24,3	1,11	
≥60	56,1	0,41		50,2	0,52		53,4	0,44		42,0	0,80		48,6	0,56	
**Radioterapia** (dias)			0,001			<0,001			0,023			0,003			0,060
≤30	9,5	Ref.		11,6	Ref.		11,4	Ref.		15,1	Ref.		10,1	Ref.	
31- 60	13,0	0,73		18,3	0,63		15,7	0,73		20,2	0,75		15,0	0,67	
≥60	77,4	0,12		70,0	0,17		72,8	0,16		64,6	0,23		74,9	0,13	

^a^Ref.=referência.

As cinco regiões seguiram os mesmos padrões quando o primeiro tratamento foi a cirurgia, em que o maior percentual ocorreu até 30 dias do diagnóstico (89,0%), com a razão de prevalência da intervenção até 30 dias do diagnóstico superior aos demais intervalos de tempo. O Sul foi a região que mais se destacou, com 88,6% das cirurgias ocorrendo até 30 dias do diagnóstico. O Norte foi a que apresentou o menor percentual (78,2%) das cirurgias realizadas antes dos 30 dias (Tabela 2).

Quando comparadas a região de tratamento e a opção pela quimioterapia, ao contrário do observado na variável “cirurgia”, o maior percentual dessas intervenções se deu a partir dos 60 dias (65,0%), com a razão de prevalência desse intervalo sendo maior que as demais. O Norte teve o maior percentual do tratamento quimioterápico (56,1%), realizado posteriormente aos 60 dias do diagnóstico, e o menor dos percentuais (23,1%), realizado antes dos 30 dias (Tabela 2). 

Setenta e um porcento das radioterapias foram realizadas posteriormente aos 60 dias do diagnóstico, por isso a razão de prevalência desse intervalo foi maior que as demais. O Norte teve a maior porcentagem a partir dos 60 dias (77,4%) e a menor das sessões realizadas até os 30 dias (9,5%) (Tabela 2). 

Embora alguns atrasos para o início do tratamento sejam evidentes, não é possível cruzar os dados de forma a relacioná-los com outras variáveis para determinar se o início tardio dos tratamentos decorreu da presença de metástase, da disponibilidade do tratamento ou de outros fatores clínicos e sociais.

## Discussão

A maioria da população brasileira teve, como preconizado pela legislação, acesso ao tratamento do melanoma maligno de pele em período inferior aos 30 dias após diagnóstico confirmado. Porém, foram averiguadas desigualdades regionais significativas no tempo até o início do tratamento, a sua maioria independentemente da modalidade terapêutica inicial. 

Nesse contexto, a cirurgia foi a principal forma de tratamento indicada em todas as regiões e apresentou os menores intervalos entre diagnóstico e tratamento. Destacou-se a região Norte, onde predominou o início tardio do tratamento, especialmente quando a indicação inicial era de quimioterapia e radioterapia.

Entre as limitações desta pesquisa, destacou-se o uso de dados secundários anonimizados provenientes do pareamento dos dados de sistemas de informação formalizados nos registros do Painel-Oncologia. Isso impediu o acesso a variáveis clínicas detalhadas, como estadiamento tumoral, comorbidades, tipo histológico e desfechos clínicos. 

Há a possibilidade de subnotificação e inconsistências nos registros, além da contemplação exclusiva de casos provenientes do Sistema Único de Saúde, fomentando maior viés de seleção. Apesar disso, a abrangência nacional, a atualização periódica e a série temporal robusta fortalecem a análise de apoio ao enfrentamento das desigualdades em saúde.

Atrasos no início do tratamento oncológico estão associados ao pior prognóstico, com mais complicações e menor sobrevida, especialmente em neoplasias com potencial metastático como o melanoma (15,16,17). Logo, é importante questionar os motivos que levariam às diferenças regionais do começo da assistência em saúde, pois podem refletir de desigualdades no acesso aos serviços especializados, infraestrutura oncológica e efetividade na regulação do tratamento oncológico no território nacional (18). 

Aspecto crucial nesse contexto é a coordenação entre diferentes especialistas, visando evitar atrasos no tratamento do melanoma. Exemplo disso se traduz nas pessoas que realizam procedimentos de biópsia e excisão com o mesmo dermatologista e que, consequentemente, têm menos atrasos em seus cuidados terapêuticos (19). 

Nos Estados Unidos, por exemplo, não há uma recomendação formal que especifique um cronograma desde a biópsia até o tratamento definitivo (20,21). Já na Europa, mantém-se a sugestão de iniciar o tratamento cirúrgico dentro do intervalo de quatro a seis semanas simultaneamente à biópsia de linfonodo sentinela (22), o que corrobora os achados de maior prevalência da cirurgia em até 30 dias no Brasil. 

Conforme o estágio da doença, resvala-se na opção dos tratamentos adjuvantes, como a imunoterapia (23,24,25). Desde 2010, novos tratamentos sistêmicos foram aprovados no contexto do melanoma maligno cutâneo: imunoterapia sistêmica, inibidores do proto-oncogene B-Raf (BRAF) de agente único, regimes de combinação de inibidores BRAF-MEK e imunoterapia intralesional envolvendo um vírus herpes oncolítico modificado (26). 

Tais tratamentos obtiveram indicações iniciais no melanoma avançado e irressecável (26). No entanto, apurou-se que essas novas estratégias oferecem melhor eficácia quando comparadas à quimioterapia. Apesar dessa informação, a quimioterapia e os imunoterápicos da classe anti-PD1 (nivolumabe e pembrolizumabe) permanecem sendo as únicas terapias sistêmicas oferecidas pelo Sistema Único de Saúde no acompanhamento desse tipo de câncer, sendo o principal agente quimioterápico utilizado a dacarbazina (27). 

No Canadá (28), foram analisadas 302 pessoas diagnosticadas com melanoma cutâneo em estágio IV, metástase presente no momento do diagnóstico. Nessa população, o tempo médio para início da imunoterapia como tratamento de primeira linha foi de 52 dias (intervalo interquartil de 30,2 a 99,0 dias). Até 2025, não foram encontrados outros estudos que relatem o uso de terapias sistêmicas alternativas como escolha inicial nesse contexto clínico.

Há, ainda, a possibilidade de combinação de quimioterapia com radioterapia. Isso pode ocorrer de forma sequencial ou concomitante, a partir do pressuposto de que a administração de quimioterapia concomitante garante um melhor controle local do melanoma, otimizando os resultados da radioterapia (20). 

Depreende-se que, uma vez que as outras terapias sistêmicas forem incluídas no repertório de tratamentos disponíveis pelo Sistema Único de Saúde, os resultados de sobrevida do melanoma no estágio IV serão mais promissores do que os com uso exclusivo de quimioterapia (20). 

Quanto à terapia direcionada, sabe-se que o melanoma é um tumor relativamente radiorresistente, visto que tem a capacidade de reparar eficazmente os danos no DNA causados pela radiação (29). Por isso, a escolha da radioterapia como tratamento de primeira linha aplica-se a casos excepcionais – por exemplo, perante a impossibilidade de realizar cirurgia ou como complemento de algumas situações em que existe o elevado risco de recidiva. Por outro lado, essa modalidade terapêutica é amplamente utilizada como tratamento paliativo do melanoma metastático (estágio IV). 

A elevada prevalência de indivíduos que realizaram radioterapia após 60 dias do diagnóstico pode ser justificada pela suposição de que teriam sido identificados já em fase avançada da doença, na qual o tratamento tem finalidade paliativa, buscando o alívio dos sintomas e o prolongamento do controle local do tumor (29).

Os achados deste estudo indicaram disparidades significativas no início do tratamento oncológico de melanoma maligno de pele no Brasil, sendo a cirurgia o procedimento mais oportuno e homogêneo entre as regiões. A quimioterapia e a radioterapia apresentaram início tardio e maior variação regional, sugerindo barreiras no acesso e possíveis atrasos assistenciais. Aponta-se também a necessidade de mais estudos acerca do investimento da imunoterapia na Saúde Coletiva. Tais evidências reforçam a necessidade de políticas públicas que garantam o cumprimento da legislação vigente e a ampliação do acesso a terapias mais modernas e eficazes no Sistema Único de Saúde.

## Data Availability

O banco de dados utilizado na pesquisa está disponível em: https://osf.io/np6b8/.
